# Men seeking counselling in a Breast Cancer Risk Evaluation Clinic

**DOI:** 10.3332/ecancer.2018.804

**Published:** 2018-01-30

**Authors:** Ana Catarina Freitas, Ana Opinião, Sofia Fragoso, Hugo Nunes, Madalena Santos, Ana Clara, Sandra Bento, Ana Luis, Jorge Silva, Cecília Moura, Bruno Filipe, Patrícia Machado, Sidónia Santos, Saudade André, Paula Rodrigues, Joana Parreira, Fátima Vaz

**Affiliations:** 1 Service of Medical Oncology, Instituto Português de Oncologia de Lisboa Francisco Gentil, Rua Professor Lima Bastos, 1099-023 Lisboa, Portugal; 2 Breast Cancer Risk Evaluation Clinic, Instituto Português de Oncologia de Lisboa Francisco Gentil, Rua Professor Lima Bastos, 1099-023 Lisboa, Portugal; 3 Molecular Pathobiology Research Unit, Instituto Português de Oncologia de Lisboa Francisco Gentil, Rua Professor Lima Bastos, 1099-023 Lisboa, Portugal; 4 Service of Urology, Instituto Português de Oncologia de Lisboa Francisco Gentil, Rua Professor Lima Bastos, 1099-023 Lisboa, Portugal; 5 Service of Dermatology, Instituto Português de Oncologia de Lisboa Francisco Gentil, Rua Professor Lima Bastos, 1099-023 Lisboa, Portugal; 6 Laboratorial Diagnosis Department, Instituto Português de Oncologia de Lisboa Francisco Gentil, Rua Professor Lima Bastos, 1099-023 Lisboa, Portugal

**Keywords:** hereditary cancer, germline mutations, genetic counselling, genetic testing, breast cancer, prostate cancer, surveillance program

## Abstract

**Background:**

Hereditary breast and ovary cancer syndrome affects both genders but little is known about the uptake of genetic services by men. The objective of this study is to characterise the male population counselled through a multidisciplinary breast/ovarian program.

**Methods:**

Descriptive analysis of male patients counselled from January 2000 to December 2015. Data in this analysis include new cancer diagnoses during prospective follow up.

**Results:**

From 4,320 families registered, 362 male patients were identified: 236 (65.2%) from hereditary cancer families (HCF) and 126 (34.8%) from non-HCF. In HCF, 121 patients (51.3%) were mutation carriers (MC): *BRCA2* – 102 (84.3%), *BRCA1* – 16 (13.2%), *CHEK2* – 1 (0.8%) and *TP53* – 2 (1.7%). Non-HCF included 126 patients: 85 (67.5%) belonged to families without pathogenic mutations or with variants of unknown clinical significance; 22 (17.5%) refused testing after counselling and 19 (15.0%) did not meet criteria for testing. Both HCF and non-HCF included patients with previous cancer diagnoses: HCF- Breast Cancer (BC) - 18; prostate cancer (PC) - 13; melanoma - 1; others - 7) and non-HCF (BC - 77; PC - 20; gastric cancer (GC) - 1; melanoma - 8; bladder cancer - 1; others - 22). From the 121 MC identified (including the *TP53* and *CHEK2* carriers), 97 patients (80.2%) adhered to prospective surveillance. With a median follow-up of 36.9 months, 17 cancers were diagnosed in 14 patients, PC being the most frequently diagnosed neoplasia (5 cases). Eleven patients (78.6%) are alive and three patients died of advanced cancer (2 with GC, 1 with disseminated adenocarcinoma).

**Conclusion:**

We observed a high adherence to counselling, genetic testing and active surveillance by men belonging to hereditary BC families. Male carriers of pathogenic DNA variants are at risk for several cancers and should be included in prospective follow-up studies.

## Introduction

Family predisposition to cancer has been a clinical, individual and social concern for a long time [1]. With increasing research in cancer genetics, multiple genes and mechanisms have been identified as being involved in inherited cancer syndromes [2]. One of these, the hereditary breast and ovary cancer syndrome (HBOCS), associated with germline mutations in *BRCA1* and *BRCA2* genes, is believed to cause approximately 10–15% of all breast cancers (BCs) [3]. The prevalence of *BRCA1* and *BRCA2* mutations in the general population is estimated to be between 1 in 500 and 1 in 1,000, respectively [4, 5]. Higher prevalence of *BRCA* mutations have been described in certain founder populations, such as the Ashkenazi Jewish and the Icelandic population [6]. Despite these low rates in the general population, pathogenic variants in *BRCA1/2* genes are the most frequent genetic alterations diagnosed in familial BC, being responsible for 3–8% of all BC cases and for 15–20% of all familial BC aggregation [3]. Other high or moderate penetrance genes (that include *CHEK2, PTEN, TP53, ATM, STK11/LKB1, CDH1, NBS1, RAD50, BRIP1* and *PALB2*) have also been described as also contributing to hereditary BC [7–11]. With the increasing use of Next Generation Sequencing and panel multigene testing, this list is increasing [2, 12–14].

Because of the high incidence of BC and the increasing awareness of the possibility of inherited cases (especially since *BRCA* testing is commercially available), the majority of patients counselled and tested in BC Risk Evaluation Clinics are female [15]. However, *BRCA1/2* carriers are also at higher risk for developing gastric and pancreatic cancer and male carriers have higher prostate cancer (PC) risk [16–19]. Skin or uveal melanoma was also associated with *BRCA* mutations, but this association is not conclusive [20]. With this knowledge and since *BRCA1/2* germline mutations have an autosomal dominant transmission, affecting both genders equally, it is expected that men increase their uptake of genetic services [15]. The literature is sparse on this matter, however, and the major focus is more on the distress and psychological needs of male patients getting genetic testing and counselling [21], than on the characteristics of male patients effectively counselled in genetics clinics. Also, little is known about the surveillance programs performed, and the outcomes and compliance of male patients included in these.

The influence of male *BRCA* carriers in pedigree analysis of potential hereditary BC families has been previously acknowledged [22]. Besides the male-to-female ratio, family size and the individual clinical history of family members (death in young age, surgical procedures for non-oncological reasons that prevent the posterior development of cancer) are factors to consider, when counselling for genetic testing in BC families [23]. Not acknowledging these may prevent the identification of high-risk individuals and their families, women and men, and their participation in specific cancer surveillance programmes. The non-systematic inclusion of men from BRCA1/2 families *in the* counselling process may also prevent the opportunity to improve health practices and to transmit cancer risk information to offspring [24].

The aim of this study is to characterise the male population counselled during the first 15 years of activity in our BC Evaluation Clinic. Men with a previous diagnosis of BC were included, from the beginning of our program, since we considered male BC as criteria for *BRCA1/2* testing, even without other cancer family history. In recent years, we also started inviting healthy men from *BRCA1/2* families for carrier testing, mostly due to high PC risk [16, 18]. Our approach has been to include *BRCA1/2* male carriers in prospective long-term surveillance programmes. Men seeking counselling for other hereditary syndromes as well as other male patients seeking counselling due to non-hereditary familial BC, were also reviewed in this study.

## Patients and methods

*Patients:* Review of all consecutive genetic and clinical medical records of male patients counselled through our programme, between January 2000 and December 2015. These records include a complete personal and medical history of the patient as well as a pedigree with cancer and genetic information of at least three generations. Most men included in our program were either referred by their physicians because they had breast or PC, or were invited through proband contact when belonging to families found to harbour genetic pathogenic variants.

*Genetic counselling*: Our criteria for DNA testing are publicly available on the Internet [25]. Briefly, genetic testing was considered in cases of at least 10% combined probability of a *BRCA1/2* mutation or BC diagnosis before 30 years of age or triple negative BC before 50 or male BC or (since 2014) high-grade serous ovarian cancer. Patients with criteria for genetic testing undergo appropriate counselling. When consenting on molecular diagnosis, they return for a post-test counselling visit, for test result disclosure and post-test management. All male carriers of pathogenic variants were offered the possibility to participate in a prospective surveillance programs. For the purposes of this paper, hereditary cancer families (HCF) are defined as families with a previous identification of a pathogenic germline variant. Non-hereditary cancer families (non-HCF) did not have a germline pathogenic variant identified.

*Molecular diagnosis*: Until 2014 mutation testing for point mutations in BRCA1 and BRCA2 genes was performed using methodologies based on heteroduplex analysis: Conformational Sensitive Gel Electrophoresis [26], [27] and Conformational Sensitive Capillary Electrophoresis [26]–[28]. After 2014 *BRCA1* and *BRCA2* molecular diagnosis was performed by next generation sequencing (NGS) using the BRCA MASTR Dx (Multiplicom, Niel, Belgium) kit and multigene testing was performed using the Trusight Cancer sequencing panel (Illumina, San Diego, CA, USA) in a MiSeq plattform (Illumina, San Diego, CA, USA) [29]. Large rearrangements in BRCA1 and BRCA2 genes were tested by multiplex ligand probe amplification (MLPA, MRC Holland) and the Portuguese founder mutation specifically tested for as previously described [30].

*Prospective follow up*: Duration of follow up was defined as the period since the post-test counselling visit until the last registered visit during study period. Data collected included patients’ demographics, reason for referral, personal clinical and family history, DNA test results and follow-up data with new cancer diagnoses and survival considered as events of interest. For male *BRCA1/2* mutation carriers (MC) the surveillance program included:

- Annual medical oncology evaluation along with complete physical examination
- Annual urology evaluation with PSA screening
- Annual dermatologic screening for suspicious skin lesions
- Specific surveillance according to the Hereditary Syndrome (e.g., Colon and prostate screening for the CHEK2 1100delC carrier and a specific protocol for TP53 carriers)
- Other clinical investigation according to new symptoms.

*Statistics*: Descriptive statistics were obtained, using Microsoft Excel, for the distribution of study variables.

*Legal and ethical issues*: All genetic records are protected with restricted access as per Portuguese law. All men consenting in genetic testing or their legal representatives signed an informed consent form that was approved by the Ethics Committee of our Institute.

## Results

### Patients characteristics

**Figure 1.**
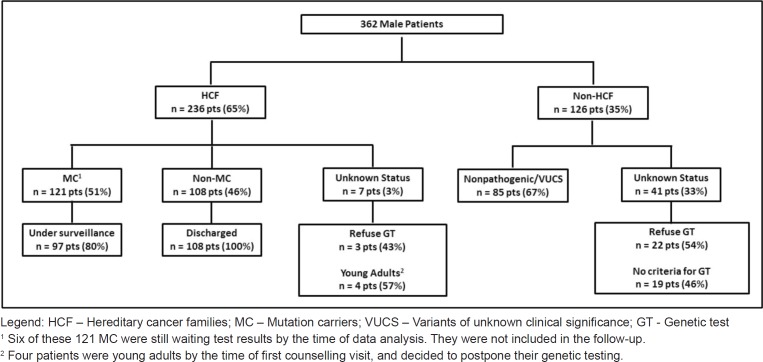
Study population.

From a total of 4,320 families registered, we identified 362 male patients, with a median age of 53 years (16–86 years). Most of them [236 patients (65.2%), median age 47.5 years] belong to HCF (Figure 1). One hundred twenty-one of these were found to be MC: *BRCA2* (102), *BRCA1* (16), *CHEK2* 1100delC variant (1) and *TP53* (2) (Table 1). Nineteen patients were the first individuals to be tested in their families, all with a previous cancer diagnosis [BC (16), PC (2), colorectal cancer (1)]. All other men in HCF were invited, through proband contact, after the identification of a pathogenic variant in a family relative. Fifty-one (50%) of all *BRCA2* carriers belong to c.156_157insAlu families, a founder mutation of Portuguese origin [30]. The non-MC of HCF was discharged from follow-up after the post-test counselling visit. Six patients in the HCF Group were waiting for test results by the time of the analysis. Their mutational status is included in the sample characterisation but they were not included in the follow-up data collection. Three patients refused genetic testing (Figure 1). They were discharged from the clinic with information to their primary care physician. The other group (Non-HCF, Figure 1) includes 126 patients (34.8%), with a median age of 63.5 years (range 1,786 years). More than half (67%) of these men belong to high risk families and were tested as index patients, but were found to have non-pathogenic mutations or variants of unknown clinical significance. Also in this group, 19 patients asked for counselling due to aggregation of BC in their families, but were found not to have criteria for genetic testing, and 22 refused testing after counselling.

**Figure 2.**
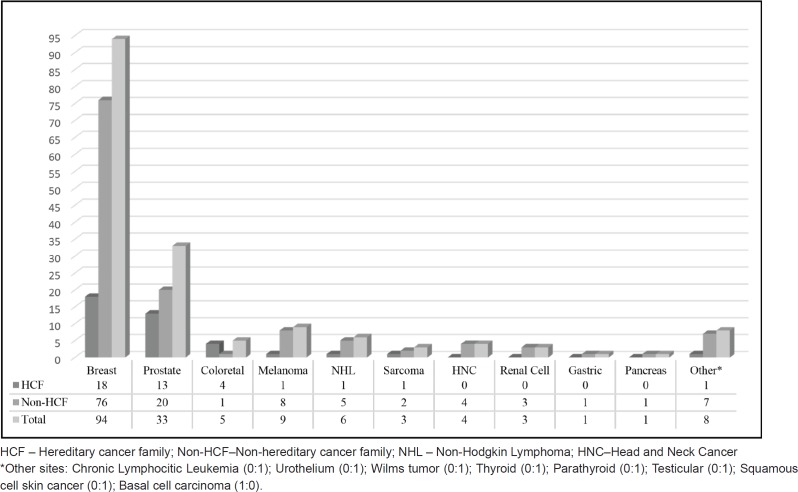
Previous cancer diagnoses.

**Figure 3.**
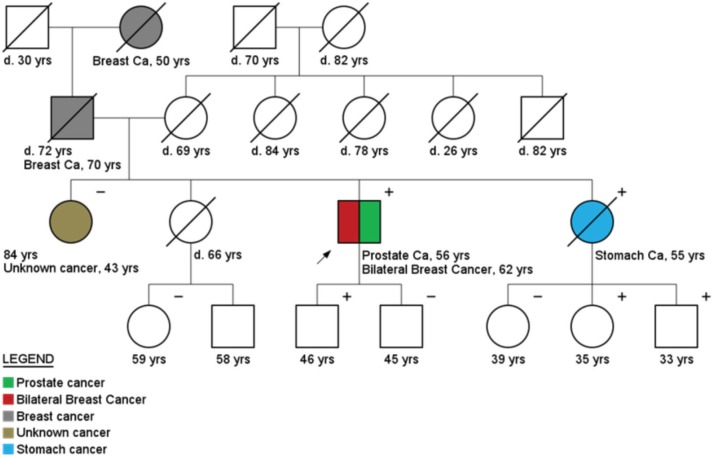
Pedigree of a bilateral breast cancer and prostate cancer patient (arrow), carrier of a BRCA2 mutation (c.9098_9099insA). Plus (+) and minus (−) signs represent the carrier status of tested family members.

**Figure 4.**
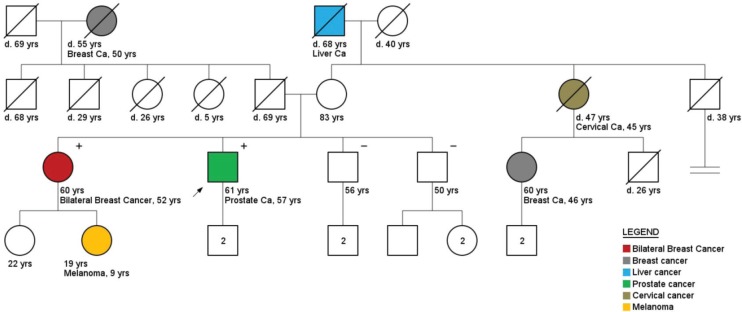
Pedigree of a prostate cancer patient (arrow), carrier of a CHEK2 mutation (1100delC). Plus (+) and minus (−) signs represent the carrier status of tested family members.

**Figure 5.**
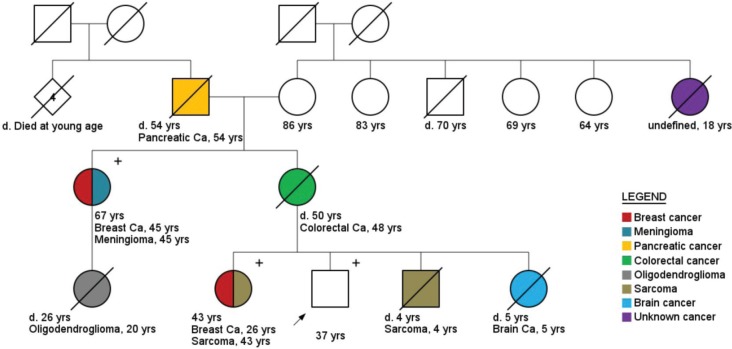
Pedigree of a male healthy carrier (arrow) of pathogenic TP53 variant (c.481G > A). Plus (+) and minus (−) signs represent the carrier status of tested family members.

Regarding cancer diagnoses before genetic testing, 33 patients belonging to HCF (33/236) had been previously diagnosed with a total of 39 cancer cases (0.17 case/patient). BC was the most frequent diagnosis (46%), followed by prostate (33%) and colorectal cancer (10%) (Figure 2). Although these individuals belonged to families with a known hereditary cancer syndrome, only 26 were confirmed carriers of the family mutation. The other seven cancer cases were admitted to be likely sporadic: the other side of the family was not suspicious for cancer heredity, and even when it was (only 1 case) comprehensive *BRCA1/2* analysis did not reveal any pathogenic variant. An example of a family with two cases of male BC and found to harbour a *BRCA2* mutation is shown in Figure 3. More complex hereditary families are shown in Figure 4 (*CHEK2 1100delC*) and Figure 5 (Li-Fraumeni Syndrome).

Male patients from non-HCF included 107 individuals with a personal history of neoplasia (129 cases; 1.02/patient). BC represented 77 of these cases (60%). Figure 2 displays both HCF and non-HCF cancer diagnoses registered before testing.

*Prospective follow up*: Ninety-seven male MC were included in this prospective surveillance program, 19 of which were cancer survivors. With a median follow-up of 36.9 months (range 0–131,1mo), 14 patients were diagnosed with 17 new cancer cases (Table 2). For those 14 patients the median follow-up was 82.6 months). PC was the most frequently diagnosed neoplasia (5 cases). Eleven patients (78.6%) are alive, all but one without relapse (this one is a patient diagnosed with advanced pancreatic cancer alive after 36 months after his diagnosis). Three patients were diagnosed with advanced cancer and died of the disease (two with gastric cancer (GC) and one with disseminated adenocarcinoma).

An example of the importance of the surveillance programs for MC is the case of a 76-year-old male BC patient (index patient indicated with an arrow in Figure 3) that started follow up in April 2005, after being diagnosed with a BRCA2 germline mutation (c.9098_9099insA). Three new early cancers were diagnosed and treated during follow up (PC, contralateral BC and Bladder cancer). The patient is alive and in complete remission of all cancers.

## Discussion

In this study, we analysed the characteristics of male patients seeking counselling in our clinic and reviewed data from prospective follow up of MC at risk for hereditary cancer. Most of our high-risk patients are carriers of *BRCA1/2* pathogenic variants and adhered to a surveillance plan that allowed for cancer detection and treatment in 14 patients. In 10 of these patients (71%) early detection led to successful treatment. In four patients (GC (2), metastasis of unknown origin (1), pancreatic cancer (1)), their cancer diagnosis was made in an advanced stage and, with the exception of the patient diagnosed with advanced pancreatic cancer (alive 36 months after his initial diagnosis), they all died of progressive disease. The unexpected survival of the patient with advanced pancreatic cancer may be related with the known platinhyper sensitivity of *BRCA* cancers [31, 32].

The predominance of *BRCA1/2* male carriers in our study (contrasting with rarer *TP53* and *CHEK2 carriers*) is related to the predicted prevalence of these different genetic events in the general population. Also, multigene panels started to be regular practice in molecular diagnosis only recently [14], and our cohort started in 2000. It is likely that the number of male carriers of other pathogenic non-*BRCA1/2* mutations will increase in the future.

The significant underrepresentation of *BRCA1* MC in our sample is due to several factors. First, there was a delay in testing and counselling male individuals from *BRCA1* families, since *BRCA2* men seemed to be at higher cancer risks and there was no clear recommendation to test men [19]. More recently, however, not only *BRCA2* but also *BRCA1* status has been considered one of the factors to take into account before deciding on PC screening [16], and we started including men from *BRCA1* families in the counselling process.

In a universe focused on female breast and ovarian hereditary cancers [15], it is important to understand how these men were referred for counselling. Besides the increased awareness for the risk of inherited cancer, the main reason for male patient referral is a previous BC diagnosis. This is particularly true in our study population, in which BC was the most frequent cancer observed among previously affected patients. It is estimated that *BRCA* mutations are responsible for nearly 10% of male BC [33], with a reported lifetime risks of inherited male BC ranging from 5% to 10% in *BRCA2* MC and 1% to 5% in *BRCA1* MC [34]. Also, it seems that the probability of a BRCA mutation (especially *BRCA2*) increases if there is a significant family history of breast and ovarian cancer [33, 35]. For PC, 2% of the cases are attributable to *BRCA* mutations and the estimated lifetime risk for PC is 20% in *BRCA1* MC and 40% in *BRCA2* MC [35, 36]. This data helps to explain why BC (94) and PC (33) were the most frequently observed cancers in previously affected patients. Either through their physician or through self-referral, these men felt at risk of belonging to a hereditary cancer family. Counselling for individuals in Hereditary Prostate Cancer Families (HPCF) is very complex, since other known and unknown genetic factors besides *BRCA1/2* mutations seem to be implied [37]. In our cohort 28 of all 4,320 families reviewed met criteria for HPCF (three or more cases of PC in first degree relatives; patients with PC in each of three successive generations or two first degree relatives with PC before the age of 55). Of these, 18 PC patients consented on *BRCA1/2* testing after counselling but *BRCA2* pathogenic variants were only observed in six cases, leaving all other HPCF without any genetic cause identified (data not shown).

Increased surveillance of healthy *BRCA1/2* male carriers is still controversial, even if higher cancer risks are recognised [19, 36, 38, 39]. Data are accumulating however that, at least for *BRCA2* male carriers, early detection is advisable: *BRCA2* male BC displays pathologic characteristics related with greater biological aggressiveness, in comparison either with the sporadic male counterpart or *BRCA1/2* female cases [40]. Also, *BRCA2* PC displays an aggressiveness that justifies treatment [41]. We observed an unexpected good compliance with follow up (82% of all carriers kept regular surveillance) and the neoplasia most frequently diagnosed was PC, with five cases of Gleason 6–9 (data not shown). Early treatment has allowed, so far, survival without relapse in all PC patients. As previously mentioned, two deaths attributable to GC were observed. On the other hand, some studies include GC in the *BRCA2* phenotype [19, 42, 43], others did not confirm this [36]. Other factors may contribute for the GC cases observed in our *BRCA2* carriers, since Portugal has a high incidence and mortality of this cancer [42]. There are no clear guidelines regarding screening of digestive cancers in *BRCA2* carriers and even for pancreatic cancer screening (part of both the *BRCA1* and *BRCA2* phenotype [33]) the recommendation is to include those carriers in prospective studies [44].

With the progressive inclusion of multigene panels in molecular testing, the number of male carriers of non-*BRCA* mutations is likely to increase, adding to the complexity of counselling and surveillance. *TP53* carriers pose a complex challenge to cancer risk management clinics [45]. This patient population not only is at high risk for multiple primary neoplasia since childhood [46], as they also have an increased susceptibility to DNA damage by ionising radiation, limiting the use of radiologic exams in surveillance [45–47]. Most ­surveillance ­recommendations for Li-Fraumeni patients and their families focus mainly on breast and colorectal cancer surveillance, which may be insufficient given the high-risk for other types of cancer such as bone and soft tissue sarcoma (diagnosed in 25% of all germline *TP53* MC [48]), brain tumours, leukaemia, adrenocortical carcinoma among others less frequently observed (gastric, prostate, pancreas, melanoma, lymphoma) [45, 47, 49–51]. Interest in the concept of “whole-body imaging” is emerging, and a number of trials assessing the role of whole-body MRI (WB-MRI) in high-risk cancer patients are ongoing [52, 53]. Early this year, the first results of the SIGNIFY study were published [54]. This was a small UK trial, aiming to evaluate the role of WB-MRI in *TP53* MC screening. Eighty-eight patients were enrolled, 44 were MC and the other 44 patients were healthy control individuals. During the study, six patients in the experimental arm had a cancer diagnosed, but only four were a direct result of WB-MRI screening. One of the questions raised by this study was the false-negative findings leading to further investigation and radiation, and the potential distress caused by these [54]. More robust data is needed before recommendations can be made.

The clinical management of patients harbouring moderate penetrance gene variants (like *CHEK2* gene) is challenging. The *CHEK2* 1100delC mutation may explain up to 5% of BC families with a *BRCA1/2* phenotype but with a *BRCA1/2* negative test result [55, p. 2]. This particular mutation has been shown to increase female BC risk by 2- to 3-fold and a 10-fold increase risk in male BC [10]. It has also been associated with PC [56]. According to NCCN guidelines, male MC for a *CHEK2* gene mutation lack specific medical management guidelines for BC risk. Nevertheless, this risk has increased the concern about male BC screening, patient breast awareness education, clinical breast examinations, and mammography [49, 57]. To include or not, prostate and colorectal cancer screening is still debatable.

For high-risk couples where the male is a *BRCA1*, *BRCA2* or a *TP53* carrier, counselling should include discussion regarding the possibility of preimplantation genetic diagnosis (PGD) [46, 58]. PGD uses fertilisation in vitro technology and allows embryos to be tested prior the transfer to the uterus, according to their mutational status [46, 58]. Although PGD implies some ethical issues that are not in the framework of this study, several couples are interested in pursuing it, and proper counselling should be made available.

Several questions remain to be answered, and one of them is the management of high-risk patients with variants of unknown clinical significance [59]. Although 18% of all BC cases tested by our group were found to be carriers of a *BRCA1/2* pathogenic mutation, nearly 30% of our male referrals because of a cancer diagnosis (including multiple previous diagnoses) had an inconclusive test result. This can be a source of distress [60] for patients and their families and poses unique problems in management and follow up since there is a lack of predictive cancer models for these men.

## Conclusion

Men from HBOCS families actively seek counselling concerning their risk for hereditary cancer, and the great majority of carriers with pathogenic variants are compliant with increased surveillance. Management of cancer risk is complex, not only for *BRCA1/2* and *TP53* MC but also for men at high risk of hereditary cancer without identified pathogenic variants. It is likely that the current use of panel testing will identify new genes responsible for Breast, Prostate and other male cancers in HBOCS like families. This may clarify the genetic contributor but will add to the complexity of clinical management. Genetics Clinics need to adapt to the needs of a growing male population and more clinical research is also needed to develop guidelines for the management of complex phenotypes.

## Conflicts of interest

The authors have no conflicts of interest to declare.

## Figures and Tables

**Table 1.** Patients’ characterisation by mutational status.

	MC	Non-MC*	Unknown^1^	Total
Patients (No)	121	193	48	362
Age at first visit (years)				
Mean	48	55	52	53
Range	23–78	16–86	16–81	16–86
Healthy (No)	95	110	17	222
Age at first visit, years				
Mean	44	46	37	47
Range	23–78	16–79	16–64	16–79
Previously affected (No)	26	83	31	140
Age at first visit, years				
Mean	68	64	58	64
Range	48–77	32–86	16–81	16–86
Previous neoplasia (No)				
1	12	68	26	106
2	10	14	4	28
3	3	0	1	4
>3	2	1	0	3
Mutations				
*BRCA2*	102			
*BRCA1*	16			
*p53*	2			
*CHEK2*	1			

MC – Mutation carriers; Non MC - non mutation carriers

* these include true negative carriers for their family mutation and index patients found with non-pathogenic variants or variants of uncertain clinical significance

1 Unknown status: these patients were not tested, either because they did not meet criteria for genetic testing or refused to be tested after counselling.

**Table 2.** Cancer diagnoses during follow-up.

FUP Diagnoses	*T* = 17
Prostate cancer	5
Gastric cancer	3
Breast cancer	2
Colorectal cancer	2
Pancreas cancer	1
Kidney cancer	1
Urothelium	1
Thyroid	1
Occult primary	1

## References

[1] Broca P (1866). Traité des tumeurs.

[2] Weitzel JN, Blazer KR, MacDonald DJ (2011). Genetics, genomics and cancer risk assessment: state of the art and future directions in the era of personalized medicine. CA Cancer J Clin.

[3] Lux MP, Fasching PA, Beckmann MW (2006). Hereditary breast and ovarian cancer: review and future perspectives. J Mol Med Berl Ger.

[4] The Anglian Breast Cancer (ABC) Study Group (2000). Prevalence and penetrance of BRCA1 and BRCA2 mutations in a population-based series of breast cancer cases. Br J Cancer.

[5] Ford D, Easton DF, Peto J (1995). Estimates of the gene frequency of BRCA1 and its contribution to breast and ovarian cancer incidence. Am J Hum Genet.

[6] Roa BB, Boyd AA, Volcik K (1996). Ashkenazi Jewish population frequencies for common mutations in BRCA1 and BRCA2. Nat Genet.

[7] Lalloo F, Varley J, Ellis D (2003). Prediction of pathogenic mutations in patients with early-onset breast cancer by family history. Lancet Lond Engl.

[8] Brownstein MH, Wolf M, Bikowski JB (1978). Cowden’s disease: a cutaneous marker of breast cancer. Cancer.

[9] Pharoah PD, Guilford P, Caldas C (2001). Incidence of gastric cancer and breast cancer in CDH1 (E-cadherin) mutation carriers from hereditary diffuse gastric cancer families. Gastroenterology.

[10] Meijers-Heijboer H, van den Ouweland A, Klijn J (2002). Low-penetrance susceptibility to breast cancer due to CHEK2(*)1100delC in noncarriers of BRCA1 or BRCA2 mutations. Nat Genet.

[11] Concannon P, Haile RW, Børresen-Dale AL (2008). Variants in the ATM gene associated with a reduced risk of contralateral breast cancer. Cancer Res.

[12] Mauer CB, Pirzadeh-Miller SM, Robinson LD (2014). The integration of next-generation sequencing panels in the clinical cancer genetics practice: an institutional experience. Genet Med.

[13] Tung N, Battelli C, Allen B (2015). Frequency of mutations in individuals with breast cancer referred for BRCA1 and BRCA2 testing using next-generation sequencing with a 25-gene panel. Cancer.

[14] Kurian AW, Kingham KE, Ford JM (2015). Next-generation sequencing for hereditary breast and gynecologic cancer risk assessment. Curr Opin Obstet Gynecol.

[15] Lang KA (2013). Genetic counseling for breast cancer risk: how did we get here and where are we going?. Expert Rev Mol Diagn.

[16] Carroll PR, Parsons JK, Andriole G (2016). NCCN guidelines insights: prostate cancer early detection, version 2.2016. J Natl Compr Cancer Netw.

[17] Couch FJ, Farid LM, DeShano ML (1996). BRCA2 germline mutations in male breast cancer cases and breast cancer families. Nat Genet.

[18] Bancroft EK, Page EC, Castro E (2014). Targeted prostate cancer screening in BRCA1 and BRCA2 mutation carriers: results from the initial screening round of the IMPACT study. Eur Urol.

[19] Liede A, Karlan BY, Narod SA (2004). Cancer risks for male carriers of germline mutations in BRCA1 or BRCA2: a review of the literature. J Clin Oncol.

[20] BRCA1 and BRCA2 families and the risk of skin cancer. https://www.ncbi.nlm.nih.gov/pubmed/20809262.

[21] Strømsvik N, Råheim M, Oyen N (2009). Men in the women’s world of hereditary breast and ovarian cancer--a systematic review. Fam Cancer.

[22] Limited family structure and BRCA gene mutation status in single cases of breast cancer. https://www.ncbi.nlm.nih.gov/pubmed/17579227.

[23] Rebbeck TR, Friebel T, Lynch HT (2004). Bilateral prophylactic mastectomy reduces breast cancer risk in BRCA1 and BRCA2 mutation carriers: the PROSE study group. J Clin Oncol.

[24] Exploring family relationships in cancer risk counseling using the genogram |cancer epidemiology, biomarkers & prevention. http://cebp.aacrjournals.org/content/8/4/393.full-text.pdf.

[25] IPOLFG (in Portuguese). http://www.ipolisboa.minsaude.pt/Default.aspx?Tag=CONTENT&ContentId=9296.

[26] Ganguly A, Rock MJ, Prockop DJ (1993). Conformation-sensitive gel electrophoresis for rapid detection of single-base differences in double-stranded PCR products and DNA fragments: evidence for solvent-induced bends in DNA heteroduplexes. Proc Natl Acad Sci U S A..

[27] Mattocks CJ, Watkins G, Ward D (2010). Interlaboratory diagnostic validation of conformation-sensitive capillary electrophoresis for mutation scanning. Clin Chem.

[28] Hill M (2011). Conformation sensitive gel electrophoresis. Methods Mol Biol.

[29] Shokralla S, Porter TM, Gibson JF (2015). Massively parallel multiplex DNA sequencing for specimen identification using an Illumina MiSeq platform. Sci Rep.

[30] Machado PM, Brandão RD, Cavaco BM (2007). Screening for a BRCA2 rearrangement in high-risk breast/ovarian cancer families: evidence for a founder effect and analysis of the associated phenotypes. J Clin Oncol.

[31] Golan T, Sella T, O’Reilly EM (2014). Overall survival and clinical characteristics of pancreatic cancer in BRCA mutation carriers. Br J Cancer.

[32] Holter S, Borgida A, Dodd A (2015). Germline BRCA mutations in a large clinic-based cohort of patients with pancreatic adenocarcinoma. J Clin Oncol.

[33] Levy-Lahad E, Friedman E (2007). Cancer risks among BRCA1 and BRCA2 mutation carriers. Br J Cancer.

[34] Evans DG, Bulman M, Young K (2008). BRCA1/2 mutation analysis in male breast cancer families from North West England. Fam Cancer.

[35] Lecarpentier J, Silvestri V, Kuchenbaecker KB (2017). Prediction of breast and prostate cancer risks in male BRCA1 and BRCA2 mutation carriers using polygenic risk scores. J Clin Oncol.

[36] van Asperen CJ, Brohet RM, Meijers-Heijboer EJ (2005). Cancer risks in BRCA2 families: estimates for sites other than breast and ovary. J Med Genet.

[37] Harris JN, Bowen DJ, Kuniyuki A (2009). Interest in genetic testing among affected men from hereditary prostate cancer families and their unaffected male relatives. Genet Med.

[38] Stadler ZK, Salo-Mullen E, Patil SM (2012). Prevalence of BRCA1 and BRCA2 mutations in Ashkenazi Jewish families with breast and pancreatic cancer. Cancer.

[39] Leachman SA, Lucero OM, Sampson JE (2017). Identification, genetic testing, and management of hereditary melanoma. Cancer Metastasis Rev.

[40] Silvestri V, Barrowdale D, Mulligan AM (2016). Male breast cancer in BRCA1 and BRCA2 mutation carriers: pathology data from the consortium of investigators of modifiers of BRCA1/2. Breast Cancer Res.

[41] Taylor RA, Fraser M, Livingstone J (2017). Germline BRCA2 mutations drive prostate cancers with distinct evolutionary trajectories. Nat Commun.

[42] Morais S, Ferro A, Bastos A (2016). Trends in gastric cancer mortality and in the prevalence of Helicobacter pylori infection in Portugal. Eur J Cancer Prev.

[43] Breast Cancer Linkage Consortium (1999). Cancer risks in BRCA2 mutation carriers. J Natl Cancer Inst.

[44] Robson ME, Bradbury AR, Arun B (2015). American society of clinical oncology policy statement update: genetic and genomic testing for cancer susceptibility. J Clin Oncol.

[45] Li-Fraumeni syndrome: cancer risk assessment and clinical management. https://www.ncbi.nlm.nih.gov/pubmed/24642672.

[46] Sorrell AD, Espenschied CR, Culver JO (2013). Tumor protein p53 (TP53) testing and Li-Fraumeni syndrome: current status of clinical applications and future directions. Mol Diagn Ther.

[47] Surveillance recommendations for patients with germline TP53 mutations. https://www.ncbi.nlm.nih.gov/pubmed/26049273.

[48] Ognjanovic S, Olivier M, Bergemann TL (2012). Sarcomas in TP53 germline mutation carriers: a review of the IARC TP53 database. Cancer.

[49] NCCN guidelines insights: genetic/familial high-risk assessment: breast and ovarian. https://www.ncbi.nlm.nih.gov/pubmed/28040716.

[50] 749-Risk management for adults with a TP53 mutation. https://www.eviq.org.au/cancer-genetics/risk-management/749-risk-management-foradults-with-a-tp53-mutatio.

[51] Familial breast cancer: classification, care and managing breast cancer and related risks in people with a family history of breast cancer. https://www.nice.org.uk/guidance/cg164.

[52] LIFSCREEN: evaluation of whole body MRI for early detection of cancers in subjects with P53 mutation (Li-Fraumeni Syndrome). https://clinicaltrials.gov/ct2/show/NCT01464086?term=lifscreen&cntry1=EU%3AFR&rank=1.

[53] ANZCTR – Registration. https://www.anzctr.org.au/Trial/Registration/TrialReview.aspx?ACTRN=12613000987763.

[54] Baseline results from the UK SIGNIFY study: a whole-body MRI screening study in TP53 mutation carriers and matched controls. https://www.ncbi.nlm.nih.gov/pmc/articles/PMC5487773/.

[55] CHEK2 Breast Cancer Case-Control Consortium (2004). CHEK2*1100delC and susceptibility to breast cancer: a collaborative analysis involving 10,860 breast cancer cases and 9,065 controls from 10 studies. Am J Hum Genet.

[56] Hale V, Weischer M, Park JY (2014). CHEK2*1100delC mutation and risk of prostate cancer. Prostate Cancer.

[57] Bevers TB, Anderson BO, Bonaccio E (2009). NCCN clinical practice guidelines in oncology: breast cancer screening and diagnosis. J Natl Compr Cancer Netw.

[58] Verlinsky Y, Rechitsky S, Verlinsky O (2001). Preimplantation diagnosis for p53 tumour suppressor gene mutations. Reprod Biomed Online.

[59] Welsh JL, Hoskin TL, Day CN (2017). Clinical decision-making in patients with variant of uncertain significance in BRCA1 or BRCA2 genes. Ann Surg Oncol.

[60] Culver JO, Brinkerhoff CD, Clague J (2013). Variants of uncertain significance in BRCA testing: evaluation of surgical decisions, risk perception, and cancer distress. Clin Genet.

